# The comorbidity of low back pelvic pain and risk of depression and anxiety in pregnancy in primiparous women

**DOI:** 10.1186/s12884-018-1929-4

**Published:** 2018-07-04

**Authors:** Rosa Virgara, Carol Maher, Gisela Van Kessel

**Affiliations:** 1 Lyell McEwin Hospital, Haydown Road, Elizabeth Vale, SA 5112 Australia; 2 Alliance for Research in Exercise, Nutrition and Activity (ARENA), School of Health Sciences & Sansom Institute for Health Research. School of Health Sciences, University of South Australia, City East Campus, GPO Box 2471, Adelaide, SA 5001 Australia; 3 School of Health Sciences and Sansom Institute for Health Research University of South Australia, City East Campus GPO Box 2471, Adelaide, SA 5001 Australia; 4 Alliance for Research in Exercise, Nutrition and Activity (ARENA), School of Health Sciences, University of South Australia, City East Campus, Playford Building Level 7 Room 02, PO Box 2471, Adelaide, SA 5001 Australia

**Keywords:** Low back pain, Pelvic pain, Depression, Anxiety, Pregnancy

## Abstract

**Background:**

Approximately 50% of Australian women experience low back pain in pregnancy, with somewhere between 8 and 36% of women suffering from pregnancy related depression/anxiety. Both low back and pelvic pain and depression and anxiety are associated with poor maternal health outcomes, including increased sick leave, higher rates of functional disability, and increased access to healthcare. It also impacts upon time and mode of delivery with an increase in inductions and elective caesarean sections. For babies of women with depression and anxiety preterm birth, low birth weight and intrauterine growth restriction are all common complications. Given these poor health outcomes, it is important to determine the co-morbidity of low back and pelvic pain and depression/anxiety in pregnancy.

**Methods:**

A cross sectional study of a hospital based sample of 96 nulliparous women were assessed at 28 weeks as part of their routine antenatal appointment. Data was collected via interview and clinical records and included the Edinburgh Depression Scale (EDS), the Numerical Rating Scale (NRS) and the Modified Oswestry Low Back Pain Disability Questionnaire (MODQ). Spearman’s correlation co-efficients, prevalence ratios and ANOVA were used to determine comorbidity.

**Results:**

96 women consented to participation in the study. All study outcomes were moderately correlated. There were three main findings: One, there was a positive correlation between low back and pelvic girdle pain (LBPP) and depression/anxiety was rho = 0.39, *p* < 0.001, between LBPP and functional disability was rho = 0.51, p < 0.001 and between risk of depression/anxiety and functional disability was rho = 0.54, *p* < 0.001. Two, a woman with LBPP was 13 times more likely to have increased risk of depression/anxiety, whilst a woman with increased risk of depression/anxiety was 2.2 times more likely to have LBPP and finally three, amongst women who reported LBPP, the level of disability experienced was significantly higher in women who had concurrent increased risk of depression/anxiety (*p* = 0.003). This occurred even though the severity of pain did not differ between groups (NRS score mean *p* = 0.38).

**Conclusions:**

This study found a high level of co-occurrence of LBPP, functional disability and depression/anxiety in women in their third trimester of pregnancy. Importantly women who reported higher depression/anxiety symptoms appeared to experience higher levels of functional disability in relation to their LBPP, than women with lower depression/anxiety symptoms and LBPP.

## Background

Approximately 50% of Australian women experience low back and pelvic pain (LBPP) during pregnancy [1]. This places the prevalence of pregnancy related LBPP in Australian women in the mid-range of international findings with rates ranging from 25 to 90% [2–7]. Whilst there is much discussion in the literature about the differentiation between pregnancy-related low back pain, pregnancy-related pelvic girdle pain and combined pain issues, all are highly prevalent. The aetiology is explained as due to a combination of hormonal changes, postural changes, reduced stability due to increasing strain on core muscles, metabolic factors, genetic factors and increased parity [4]. The consequences of pregnancy-related low back pain include increased sick leave, higher rates of functional disability, and increased access to healthcare for symptom management. It has also been shown to impact upon time and mode of delivery with an increase in inductions and elective caesarean sections [1, 5, 7].

Depression and/or anxiety are also common complications of pregnancy, with international prevalence rates ranging between 8 and 36% [8–18]. The experience of depression and/or anxiety tends to peak in the third trimester, with rates as high as 21.6% for depression and 35.8% for anxiety [16]. Antenatal depression and/or anxiety has been consistently linked with shorter duration of pregnancy, increased induction rates, increased surgery and instrumental births, preterm birth, low birth weight and intrauterine growth restriction [8, 13, 14]. The latter three have been shown to follow on to be leading causes of morbidity, mortality and developmental issues in neonates, infants and children worldwide [8]. Antenatal depression and/or anxiety is a strong predictor of post-natal depression [9]. Women with antenatal depression and/or anxiety have also been found to have more somatic complaints and sick leave [10, 11].

There is limited evidence regarding the co-morbidity of low back and pelvic girdle pain and depression and/or anxiety in the antenatal period. Most work focuses on the post-natal period. However, Bakker et al. [19] examined psychological determinants of pregnancy-related lumbo-pelvic pain, using a longitudinal study examining women at 12, 24 and 36 weeks gestation. They found that there was a strong correlation between back pain and overall complaints *R* = 0.743, *p* < 0.001 at 36 weeks gestation [19]. They also found that lumbo-pelvic pain was significantly associated with perceived stress scale and pregnancy related anxiety questionnaire in all stages of pregnancy. Robinson et al. [20] conducted an earlier study in which the risk factors in early pregnancy were compared to disability or pain intensity in late pregnancy. They found that 50% of their sample had some form of pelvic pain (anterior, posterior or both), that disability rating index scores and pain intensity increased from early pregnancy (14 weeks) to late pregnancy (30 weeks) and there was a statistically significant relation between pain intensity and disability rating index scores (*r* = 0.63, *p* < 0.001) [20]. They also found that high scores on The Hopkins Symptom Check List (which measures anxiety, depression and somatisation) were significantly associated with high scores for disability rating index at 30 weeks gestation.

To date, evidence suggests that both anxiety and/or depression and low back and pelvic girdle pain have important implications for maternal and foetal health, however, there is limited research examining their co-occurrence.

## Methods

### Aims

The aims of this study were to one, examine the relationships between low back and pelvic girdle pain (LBPP), risk of depression and/or anxiety and LBPP-related disability in pregnancy; two, determine how the presence of one impacted the likelihood of the other; and three, whether prevalence of increased risk of depression/anxiety was related to the experience of LBPP-related disability.

### Design

A cross sectional study was conducted between December 2009 and February 2010. Ethics approval was obtained from the University of South Australia Human Research Ethics Committee and Central Northern Adelaide Health Service Ethics of Human Research Committee.

### Setting

The study was conducted at the antenatal clinics at the Lyell McEwin Hospital, a tertiary hospital, in the Northern suburbs of Adelaide, South Australia, in a low socio-economic area. The annual birth rate in 2009 was 3050 births. These clinics include a combination of both midwife-led antenatal care in a birthing unit with a minimalist intervention approach, and traditional doctor-led antenatal care in a labour ward.

### Participants

Data collection was undertaken across an eight-week period. Women were recruited between December 2009 and January 2010, from a consecutive hospital-based sample (i.e. every patient presenting to the hospital meeting the inclusion criteria was invited to participate until the required sample size was achieved). The inclusion criteria required that the women were currently pregnant (28/40), nulliparous, capable and willing to give informed consent, and were a current patient in the Lyell McEwin Hospital antenatal clinics. There were no inclusion/exclusion criteria based on age. Women were assessed at 28 weeks gestation as there is a higher prevalence rate (both period and point) of LBPP, as well as risk of depression/anxiety in the third trimester [21, 22]. Nulliparous women were recruited to eliminate the effect of previous pregnancy experiences on outcome measures.

**Fig. 1:**
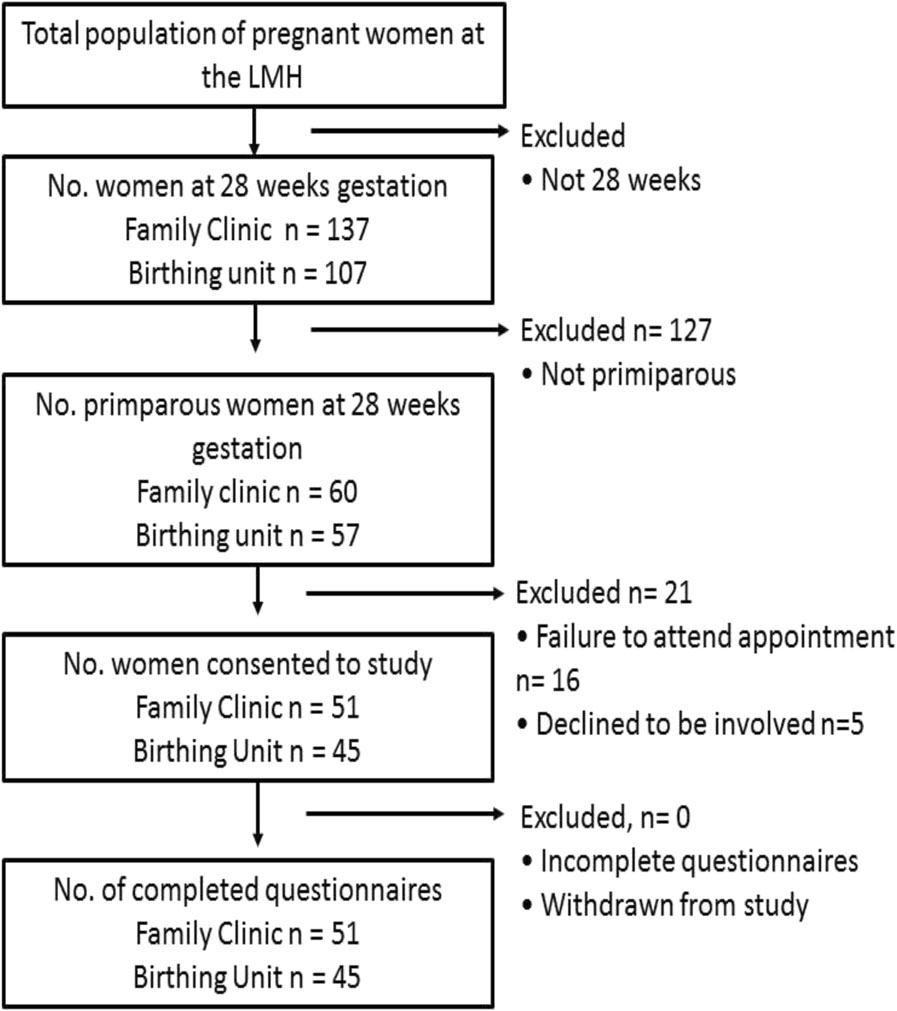
Flowchart of Sample Derivation

Case notes of all women booked in for 28-week appointments were screened, and all potentially eligible women were verbally invited to participate by the study personnel. Testing coincided with the women’s routine antenatal appointment which allowed for non-English speaking women to participate, as their interpreter was present and could interpret the participant information sheet, protocol, tools and ensure informed consent. All consecutive women meeting the inclusion criteria were invited to participate. Verbal and written consent was obtained prior to enrolment in the study. A summary of this is process is outlined in Fig. 1 below.

Demographic details (age, BMI, previous pregnancies, history of mental health issues) were gathered from participants’ case notes by study personnel. Two survey items collected previous history of low back and pelvic girdle pain prior to pregnancy, and the use of pain medication prior to their appointment. History of mental health issues were captured from the case notes, on the basis of either, one, the participant having case note records for medical treatment sought through the Lyell McEwin Hospital for mental health issues, or two, if patients reported a history of mental health issues to the midwife during their first antenatal visit (for example, depression, anxiety, bipolar disorder, bulimia etc). Women were then required to answer two questions about previous history of low back and pelvic girdle pain prior to pregnancy, and the use of pain medication prior to their appointment.

### Outcome measures

The intensity and location of LBPP was assessed using the Numerical Rating Scale and a pain body chart. The Numerical Rating Scale (NRS) is a valid measure of pain intensity on an interval scale with zero (0) indicating no pain and ten (10) indicating the worst pain [23]. The NRS has been demonstrated to be more reliable in patients that are both literate and illiterate with *r* = 0.96 and 0.95 respectively [23]. It has higher sensitivity than other similar pain intensity scales, such as Verbal Rating Scale and similar sensitivity to the Visual Analogue Scale [24]. Patients were asked to depict the location of pain on a body chart. For the purpose of this study, LBPP was defined as a score of > 0 on the NRS, coinciding with shaded areas on the body chart indicating the pain was located between the twelfth rib and inferior gluteal folds and/or the posterior iliac crest and the gluteal fold [25].

The Edinburgh Depression Scale (EDS) is a self-report measure, used to screen women during the antenatal and postnatal period for symptoms of depression and anxiety. The scale consists of 10 items and with a score range of 0–30. A high risk of depression and/or anxiety is a score ≥ 13 [26]. There is good test–retest reliability for EDS scores between 24 and 36 weeks of pregnancy (*r* = 0.63) [27]. It has high level of validity in comparison to other risk of depression screening questionnaires, with good sensitivity (0.83–0.88), specificity (0.70–0.96) and high positive predictive value [28]. Those with a score greater than 13 were referred to as having increased risk of depression/anxiety and those with a score equal to or less than 12 were referred to as not having increased risk of depression/anxiety.

Impairment of physical function was measured using the Modified Oswestry Low Back Pain Disability Questionnaire (MODQ). This scale contains 10 items measuring the degree to which back pain impacts function in a variety of aspects of daily life (such as personal care, sleeping and social life). Each item is scored from 0 to 5, with the overall scale score calculated by adding the section scores, dividing by 50 and multiplying by 100, to obtain an overall percentage of impairment [28]. Scores are interpreted as minimal disability (0 to 20%); moderate disability (21 to 40%); severe disability (41 to 60%); crippled (61 to 80%) and bed bound (81 to 100%) [29]. The MODQ has high test-retest reliability and inter-rater reliability, with intraclass coefficients (ICC) of 0.86 (95% CI 0.63–0.93) [29]. Sensitivity of 0.91 and specificity of 0.83 indicate high levels of content validity [30].

A number of steps were taken to minimise the risk of bias. Firstly, the consecutive sampling strategy and low participant burden (i.e. survey completed at medical appointment) helped ensure a high participation rate, and thus minimise selection bias. Women who could not speak English were included because an interpreter was present. By timing the study with the routine 28-week appointment, it allowed for the participation of women who were enrolled in the standard midwife-led program, standard doctor led care, as well as women who were enrolled in a shared-care model (where most pre-natal care is provided by a general practitioner, but the women attend hospital for key appointments and to give birth). Key outcomes (pain, risk of depression and anxiety, and pain-related disability) were collected using widely used tools with established reliability and validity.

Post hoc power analyses conducted using G*Power software (version 3.1.9.2, Universitat Kiel, Germany), revealed that the achieved sample size (*n* = 96) had 99% power to detect a correlation between LBPP and depression/anxiety risk of rho = 0.4, suggesting the sample size was suitable to address the research questions.

### Data analysis

This protocol resulted in a dataset with no missing data, thus imputation was not required. Spearman’s *(rho)* correlation coefficient was used to examine the relationship between risk of depression/anxiety, severity of LBPP and physical function. For the remaining analyses, risk of depression/anxiety and LBPP were dichotomised as follows: EDS was converted into no depression/anxiety, < 13 (0), and depression/anxiety ≥13 = (1). The Numerical Rating Scale was converted into no pain = 0 (0), and pain ≥1 (1).

Combined categories were created based upon risk of depression and/or anxiety and LBPP status, as demonstrated in Table 1 below:

- Group A: Women with increased risk of depression/anxiety AND LBPP
- Group B: Women without increased risk of depression/anxiety AND LBPP
- Group C: Women with increased risk of depression/anxiety AND without LBPP
- Group D: Women without increased risk of depression/anxiety AND without LBPP

**Table 1:** Combined category grouping

	EDS Score ≥ 13 (increased risk of depression)	EDS Score ≤ 12 (no increased risk of depression)
NRS > 0	A	B
NRS = 0	C	D

Prevalence ratios were then calculated to determine how likely a woman with LBPP was to have a depression/anxiety compared to a woman with no LBPP.

Prevalence ratios (PR) were calculated as follows:

$$ \mathrm{PR}=\frac{A/\left(A\pm C\right)}{B/\left(B+D\right)} $$

And how likely a woman with increased risk of depression/anxiety was to have LBPP compared to a woman with no increased risk of depression/anxiety.

$$ \mathrm{And}\ \mathrm{PR}=\frac{A/\left(A+B\right)}{C/\left(C+D\right)} $$

Finally, amongst women experiencing LBPP, ANOVA was used to examine whether risk of depression anxiety status was related to LBP disability and severity of LBPP. ANOVA was used to examine whether risk of depression anxiety status (dependent variable) was related to LBP disability and severity of LBPP (independent variables). Age and BMI were included as covariates because preliminary analyses suggested that they were associated with the independent and dependent variables using the conservative cut-off of *p* < 0.2. All analyses were conducted in IBM SPSS version 22.0 (IBM Corp, Armonk NY). An alpha of < 0.05 was used to denote statistical significance.

## Results

Data collection took place between December 2009 and February 2010. 117 women were nulliparous and thus potentially eligible for participation in the study (of the 244 pregnant women scheduled for appointments at the Lyell McEwin Hospital in this time). However, five declined to participate, and 16 failed to attend their appointment. Consequently, 96 women were recruited. The mean age was 24.5 years (SD 5.4). Mean gravidity was 1.46 (SD 0.8) indicating that while none of the women had given birth, some had a previous pregnancy not resulting in a birth. Demographic statistics and descriptive statistics for depression/anxiety risk, LBPP severity, and LBPP-related disability are reported in Table 2. There was no missing data from the sample.

**Table 2:** Participant demographic and descriptive statistics (*n* = 96)

Outcome measure	n (%)	Mean (SD)	Range
Maternal age (years)		24.5 (5.4)	15–38
Body Mass Index (kg/m2)		26.4 (6.19)	17.6–48.3
Weight status			
< 18.5, underweight	3 (3.1%)		
18.5–24.9, normal weight	43 (44.9%)		
25.0 to 29.9, overweight	25 (26.0%)		
≥ 30.0, obese	25 (26.0%)		
Gravida (no. pregnancies)		1.5 (0.8)	0–4
History of low back pain			
No	67 (69.8%)		
Yes	29 (30.2%)		
History of mental health issues			
No	91 (85.6%)		
Yes	5 (4.2%)		
Severity of LBPP (out of 10)		1.82 (2.17)	0–8
Depression/anxiety risk score (out of 30)		5.77 (4.83)	0–20
Low Risk (< 13)	82 (85.4%)		
High risk (≥13)	14 (14.6%)		
LBPP disability score (out of 100)		15.94 (12.95)	0–58
Minimal disability (0–20)	69 (71.9%)		
Moderate disability (> 20–40)	21 (21.9%)		
Severe disability (> 40–60)	6 (6.3%)		
Crippled (> 60–80)	0		
Bed bound (> 80–100)	0		

All study outcomes were moderately correlated. Specifically, the correlation between LBPP and risk of depression/anxiety was rho = 0.39, *p* < 0.001, between LBPP and functional disability was rho = 0.51, *p* < 0.001 and between risk of depression/anxiety and functional disability was rho = 0.54, *p* < 0.001.

Combined categories of increased risk of depression/anxiety and LBPP were created, and are presented in Table 3. Prevalence ratios were used to examine the likelihood of a woman having increased risk of depression/anxiety if she also had antenatal LBPP (compared to women without LBPP), and vice versa. Results revealed that a woman with LBPP was 13 times more likely to have increased risk of depression/anxiety, whilst a woman with increased risk of depression/anxiety was 2.2 times more likely to have LBPP.

**Table 3:** Combined categories of risk of depression/anxiety and LBPP pain severity

	EDS Score ≥ 13 (high risk of depression/anxiety)	EDS Score ≤ 12 (low risk of depression/anxiety)
NRS > 0	A = 13 women	B = 35 women
NRS = 0	C = 1 woman	D = 47 women

Finally, ANOVA was used to explore the experience of disability and intensity of LBPP, depending on depression/anxiety risk status as illustrated in Table 4. Amongst women who reported LBPP, the level of disability experienced was significantly higher in women who had concurrent risk of depression/anxiety (*p* = 0.003). This occurred even though the severity of pain did not differ between women with LBPP and depression/anxiety, and those with LBPP without depression/anxiety (NRS score mean *p* = 0.38).

**Table 4:** ANOVA results: examining the relationships between LBPP and disability, according to depression and anxiety risk

	Depression and anxiety risk status		F	ANOVA results: F, p
	Low	high		
LBP disability: mean, (SD), 95% CI	18.69 (11.25) 14.82–22.55	32.46 (13.93) 24.04–40.88	10.00	0.003
LBPP severity: mean, (SD), 95% CI	3.46 (1.42) 2.97–3.95	4.12 (2.20) 2.79–5.44	0.80	0.38

*Analyses adjusted for age and BMI

## Discussion

Key findings of this study were that LBPP and increased risk of depression/anxiety in pregnancy, LBPP and functional disability and increased risk of depression/anxiety and functional disability all tended to coincide. Women with LBPP were 13 times more likely to have increased risk of depression/anxiety. Amongst women with LBPP, those with increased risk of depression/anxiety had higher levels of functional disability than those without increased risk of depression/anxiety, even though the severity of their pain was the same.

The finding that the severity of LBPP and functional disability were positively correlated is not surprising. In our study, symptoms were measured at 28 weeks gestation, however, findings coincide with findings from Robinson et al. [20] and Bakker et al. [19], both of whom found that pain intensity and disability were positively correlated at 30 weeks and 36 weeks gestation respectively. Whilst each study has used different outcome measurement tools, each yielded similar results. This adds to the body of evidence demonstrating across multiple studies that pain intensity and functional disability are positively associated in pregnant women in their third trimesters.

More importantly, our study highlighted that women who had anxiety/depression in combination with LBPP experienced greater functional disability than those with LBPP alone. This occurred even though the severity of LBPP itself was similar between groups. This suggests that the presence of increased risk of anxiety/depression may change the experience and impact of LBPP. This concurs with evidence from outside the maternal care field. For example, Arnow et al. [31] examined the associations between depression, chronic pain and disability in over 5000 patients attending primary care clinics, and found that 41% of participants with depression reported disabling chronic pain, versus 10% of those without depression.

Findings highlight the importance of disability in the relationship between pain and depression. Being cross-sectional, the direction of these relationships is not clear. It is possible that pregnant women experiencing disabling LBPP are more likely to become depressed, or alternatively, that women who have an increased risk of depression/anxiety are more likely to become disabled by pain.

Strengths of the study include its high response rate and that it was conducted in an at-need setting. The hospital centre is located in a low socioeconomic setting, which serves a predominately disadvantaged population, with relatively high health care needs compared to the general Australian population. By timing testing with patients scheduled appointments, this reduced time barriers and took advantage of interpreters being present for non-English speaking participants. Another strength was the use of widely-used and validated tools to measure the key study outcomes (LBPP, functional disability and depression/anxiety). Furthermore, the research question was clinician-driven, rather than researcher-driven. As already noted, limitations included the cross-sectional study design, meaning that causality cannot be determined. Only those patients who were receiving treatment from the public health system were involved in this study, thus no data could be collected on private obstetric patients. No data was collected on patients who declined to participate in the study, therefore it is not possible to analyse for the possibility of non-responder bias. Participants with literacy difficulties were offered assistance to complete the survey; however, it is possible that such participants may have declined participation rather than accept this assistance for reasons of embarrassment. Whilst the response rate within this particular service was high, the study was conducted at one hospital, therefore it is unclear whether findings are generalizable. However, given that the study is focused on examining the *relationships between* LBPP, functional disability, and risk of depression/anxiety, findings are likely to be similar in other settings. Further research to confirm this is warranted.

### Clinical implications

This study originated from a clinical observation, that many of the women accessing ante-natal physiotherapy treatment for LBPP were also seeing the perinatal mental health midwives. While it was clear from the literature at the outset that depression/anxiety and LBPP are indeed related, this study has added to the evidence base, by examining the three-way relationship between depression/anxiety, LBPP and disability.

The bio-psychosocial model of pain management, acknowledges that emotional distress will impact physical symptoms [32]. The model of management highlights the need to address both emotional and physical needs. The benefits of this approach have been demonstrated in the general population with low back pain. For example, Woby et al. [33] investigated the cognitive factors which were related to people who experience chronic low back pain. They found that by addressing certain cognitive behaviours, such as functional self-efficacy and catastrophizing behaviours, participants had less severe pain and less functional disability ([33] p.873–32).

The current study was completed in a major hospital in a low SES setting. It is known that people in low SES settings have higher levels of anxiety and are poorer at accessing health care, thus contributing to poorer health outcomes.

Findings of the current study suggest that such a model may be beneficial for many pregnant women experiencing LBPP. In order to better serve the needs of these patients, multi-disciplinary clinics involving perinatal mental health midwives and doctors and physiotherapists could be offered. Whilst the current study examined LBPP, depression/anxiety and disability specifically in the third trimester, such a service could potentially be beneficial in the post-natal periods as well. Such a service is likely to improve pregnant women’s pain, disability, and emotional health, and is also likely to benefit their babies’ health, given the wide-ranging impact of these conditions on perinatal events and foetal outcomes.

## Conclusions

In conclusion, this study found a high level of co-occurrence of LBPP, functional disability and depression/anxiety in women in their third trimester of pregnancy. Importantly, women who reported higher depression/anxiety symptoms appeared to experience higher levels of functional disability in relation to their LBPP, than women with lower depression/anxiety symptoms and LBPP. In the future, multi-disciplinary services incorporating musculoskeletal care, mental healthcare, and pregnancy health care, may help to better address the needs of pregnant women.

## Abbreviations

EDS: Edinburgh Postnatal Depression Scale
LBPP: Low Back Pelvic Girdle Pain
MODQ: Modified Oswestry Disability Questionnaire
NRS: Numerical Rating Scale
